# Supplementary material for: Macular pigment changes after cataract surgery with yellow-tinted intraocular lens implantation
Source: PLoS One. 2021 Mar 25;16(3):e0248506. doi: 10.1371/journal.pone.0248506 (PMC7993776; doi:10.1371/journal.pone.0248506)
Supplement: Attachment — Submitted filename: Response to Reviewers(PLOS).docx [file pone.0248506.s002.docx]

February 20^th^, 2021

Dear reviewer #1

We appreciate your immensely thorough review with positive comments and useful suggestions. We have revised our manuscript according to your suggestions. The following details are our point-by-point responses to your comments. The main revised parts are indicated in the proofreading file in red.

1) *Adding a schematic of the absorbance spectra the ocular media would be beneficial (cornea, lens, retina [e.g. MP and other chromophores]) to help readers understand the region of high-energy, short-wavelength light*

Thank you for your comment. We added figures of spectral transmission of human crystalline lenses of different age and clear and yellow-tinted IOLs. The maximum absorption wavelength of macular pigments is 460 nm and this information was added in Figure 1B. *2)The authors have correctly pointed it out but direct comparisons of RRS and 2-wavelength AF and HFP are inappropriate. Differences in measured MPOD among the 3 are difficult to assign meaning to.*
We consider that this comment is in response to the discussion from line 243 to 256.

Both two wavelength AF and HFP measure absorbance of MP, i.e. both methods measure the same thing. Therefore, the values measured by two methods should be identical, but actually there is some difference in the absolute values, although the values by two methods show high correlation. In this discussion, we compared not the absolute values but the increase rates between our study and Nolan’s study and we think this comparison was reasonable.

However, we attributed the different increase rates in two studies to the difference in the initial MP absolute values. This discussion might have some bias. Therefore, we added the following sentence in this discussion, “However, the measurement techniques used to calculate the initial MP values differed in the two studies, therefore, we should take into consideration of the difference in MP values, although the differences were thought to be minor.”  *3) Please provide spectral transmission of the SN60 WF, XY1, YP2.2 and PCB00V IOLs. This might help the reader understand the absorption contribution relative to the other ocular components*
Thank you for your suggestion. We added the spectral transmission of clear and yellow-tinted IOLs in Figure 1B.

*4) Although the author states that "No L/Z supplementation was utilized, a dietary questionnaire control might be useful*
Thank you for your comment. We asked the patients at the time of every examination and confirmed that they didn’t start taking supplements including lutein and zeaxanthin. We added the following sentence, “It was confirmed verbally that no patients started to take supplements containing lutein and/or zeaxanthin after surgery.”

*5) Tbl 2 and 3 show that virtually all of the MPOD and MPOV values increased at every position. As MP cannot be synthesized de novo and must be obtained through diet, I'm interested to learn more about the etiology. I ask out of clinical interest because the theory posited in Lines 74-79 states that increased transmission of SW light leads to decreased MP but the theory described in Lines 233-239 state that increased retinal irradiation following cataract surgery leads to enhanced retinal capture of circulating L/Z due to increased isomerization. Should all ECCE patients be using L/Z supplementation?*
Thank you for your comment. The mechanisms of increase in MP after surgery is unknown. But considering the autoregulatory system of humans, this result seems reasonable. Nolan et al speculated that “the increased visible light irradiation of the retina after cataract surgery could stimulate enhanced retinal capture of circulating lutein and zeaxanthin, perhaps due to a mechanism involving increased isomerization of the MP constituents under light exposure” as described in Discussion. We have no evidences to support their speculation. We think that whether MP increase or decrease depends on the extent of increased irradiation. When the irradiation is excessive (for example, in case with clear IOL implantation), consumption of MP may be higher than MP accumulation, and MP decreases.

*6) Lines 127-129 discussed analysis of RRS and MPOV @ 1.5 degrees measured through FAF. Comparison of these values may be inappropriate due to technique differences.*
RRS uses a different principle from AF. It measures the Raman signal of carotenoids in MP in the area of a diameter of 1 mm (= 1.6̊ ). Therefore, we compared the increase rate of MP in the similar area, i.e. MPOV within 1.5̊ eccentricity in AF and total Raman signals within 1.6̊ eccentricity. Since we compared not the absolute values but the rate of values, we consider this comparison is reasonable.
